# Error-related cardiac response as information for visibility judgements

**DOI:** 10.1038/s41598-018-19144-0

**Published:** 2018-01-18

**Authors:** Marta Łukowska, Michał Sznajder, Michał Wierzchoń

**Affiliations:** Consciousness Lab, Institute of Psychology, Jagiellonian University, Cracow, 30-060 Poland

## Abstract

Interoception provides information about the saliency of external or internal sensory events and thus may inform perceptual decision-making. Error in performance is an example of a motivationally significant internal event that evokes autonomic nervous system response resembling the orienting response: heart rate deceleration, increased skin conductance response, and pupil dilation. Here, we investigate whether error-related cardiac activity may serve as a source of information when making metacognitive judgments in an orientation discrimination backward masking task. In the first experiment, we found that the heart accelerates less after an incorrect stimuli discrimination than after a correct one. Moreover, this difference becomes more pronounced with increasing subjective visibility of the stimuli. In the second experiment, this accuracy-dependent pattern of cardiac activity was found only when participants listened to their own heartbeats, but not someone else’s. We propose that decision accuracy coded in cardiac activity may be fed as a cue to subjective visibility judgments.

## Introduction

Researchers are increasingly convinced that visual perception is in fact a multimodal process^1^. Therefore, conscious visual experience is not solely based on the quality of visual stimuli^2^. Information from other sensory modalities influences conscious visual perception, especially when input quality is poor, i.e. under a perceptual uncertainty. It has been shown that during binocular rivalry, auditory^3^, olfactory^4^, and tactile^3,5,6^ information all impact conscious visual experience. However, both exteroceptive and bodily signals may be integrated into conscious visual perception. Using continuous flashing suppression^7^, it has been demonstrated that both proprioceptive^8^ and interoceptive information^9^ affect conscious visual experience. The integration of multimodal exteroceptive information is enabled by subcortical mechanisms and cortical connectivity, whereas exteroceptive and interoceptive information are integrated^2,10^ thanks to reciprocal connections between the autonomic and central nervous systems^11^. Apart from the direct integration of visual and interoceptive signals, long-lasting changes in interoceptive states (e.g. arousal level) might also influence visual perception^12,13^. Since interoception indicates the current ‘physiological condition of a body’^14^, it affects visual experience by informing about the salience of a sensory event^15^. In other words, changes in interoceptive states inform a perceiver whether a visual event that has just occurred is meaningful^16^ and might serve as a cue while perceptual decisions about the event are made. It has been shown that interoceptive states do indeed influence conscious visual perception^17,18^ and that conscious visual perception evokes changes in interoceptive states^19,20^.

A backward masking task is another method for investigating visual perception and decision-making under great uncertainty. In such a task, an above-chance level of detection or discrimination performance is frequently observed even below the subjective threshold of awareness^21^. So, inferring awareness solely from an objective performance level does not seem to reflect the subjective accessibility of a stimulus^22^. However, judgments that are based exclusively on subjective reports are not sensitive and might result in diagnosing an absence of awareness even if participants are indeed aware^23^. It has been suggested that only metacognitive accuracy—the correspondence between subjective judgments (e.g. visibility or confidence rating) and objective performance (e.g. discrimination accuracy)—allows estimation of whether visual stimuli were experienced consciously or not^24,25^. Consequently, if we want to study how interoceptive information affects conscious visual experience, we need to consider the entire perceptual decision-making process, including both objective stimulus discrimination (Type 1 perceptual decision) and subjective awareness judgement (Type 2 perceptual decision). Moreover, it is important to recognize the stage of perceptual decision-making that is most influenced by interoceptive information.

Contrary to the classic perceptual decision approaches, such as the signal detection theory^26,27^ or ballistic accumulation model^28^, recent studies suggest that subjective awareness judgements (Type 2 perceptual decision) rely not only on the quality of visual sensory input, but also on additional sources of information, such as Type 1 motor response^29^, amount of sensory noise^30^, or Type 1 response-related processes^25^. The aforementioned studies suggest that additional information is either accessible independently, or after a Type 1 decision is made. Here, we suggest that one type of information that is uniquely available after a Type 1 response is error-related interoceptive states such as heart rate deceleration, increased skin conductance response^31^, or pupil dilation^32^. The typical pattern of autonomic nervous system response to an error resembles an orienting response^20^. Arguably, an orienting response to an error that is a motivationally significant internal event^33^ informs participants about Type 1 performance, consequently allowing them to accurately judge their performance with Type 2 ratings. Thus, we propose that error-related interoceptive states might serve as internal feedback^34^ about performance accuracy.

There is already some evidence that error-related interoceptive states may serve as a source of information for metacognitive judgements. Firstly, confidence predicts pupil dilation (used as a physiological index of uncertainty) in a two-alternative forced-choice auditory version of the random-dot motion task^35^. It has been demonstrated that in the pre-Type-1 perceptual-decision period, confidence negatively predicted the pupil dilation response: the lower the confidence, the greater the pupil dilation during the decision period. Lempert and colleagues concluded that the relation may be driven by error-related pupil dilation^36,37^. Secondly, an EEG study has shown that positivity error (Pe)—an event-related potential component associated with error detection^38^—reflects accumulated evidence that an error has been made^39^. Importantly, it has been proposed that error-related interoceptive states might contribute to Pe amplitude^33^. Hajcak^31^ supported this assumption by observing in the modified Stroop task that the greater the positivity error amplitude, the larger the detected error-related skin-conductance response. Additionally, the Pe amplitude predicts confidence rating^40^. Therefore, we predict that error-related interoceptive states predict metacognitive judgements.

Although several studies have already suggested that there is interoceptive information about decision accuracy coded in cardiac activity following the decision (i.e. heart rate deceleration could be observed after an error, as compared to correct decisions^31,41–43^), none of these studies investigated how the accuracy-dependent difference in response-related cardiac activity might be used as a cue when making metacognitive judgments (e.g. rating stimuli visibility). Moreover, none of the studies investigated response-related cardiac activity in near-threshold perception. Although, in one study error-related deceleration was found in a perceptual task, the stimuli used in the task were above threshold^41^. In the rest of the studies, tasks traditionally used in studies on cognitive control were administered: speeded modified Flanker task^43^, Go/no Go task^42^, and modified Stroop task^44^. Therefore, our study aims to replicate previous findings that suggest there is an accuracy-dependent pattern of response-related cardiac activity. Moreover, the purpose of the study is to verify whether the same effects could also be found in near-threshold visual perception. Finally, we further investigate whether interoceptive states following stimulus discrimination (Type 1 perceptual decision) might serve as information when judging stimulus visibility (i.e. making Type 2 perceptual decision).

We focused on one interoceptive process, namely cardiac activity^17,18^. In the set of two experiments, we tested whether there is an accuracy-dependent pattern of cardiac activity that may serve as information in the evidence accumulation process. Additionally, we also tested whether one may influence this pattern by delivering auditory feedback about cardiac activity and how it would affect visibility judgement and metacognitive accuracy (Experiment 2). We delivered two types of feedback in the within-subject design: real and fake. We assumed that the former would increase the accessibility of error-related interoceptive information^45^, while the latter would introduce noisy interoceptive information and mislead participants^46,47^. Therefore, we expected that if Type 1 response-related interoceptive states contribute to metacognitive judgements (i.e. Type 2 response), increasing the accessibility of interoceptive information by delivering real cardiac feedback would inform about Type 1 performance accuracy and, as a result, improve metacognitive accuracy. On the other hand, introducing noisy interoceptive information by delivering fake cardiac feedback should hinder the possibility of using Type 1 response-related interoceptive states when making metacognitive judgments.

Summing up, we hypothesise that: (1) the pattern of cardiac activity following stimulus discrimination differs with respect to discrimination accuracy; (2) auditory cardiac feedback changes the accuracy-dependent pattern of cardiac activity following stimulus discrimination.

## Results

**Figure 1:**
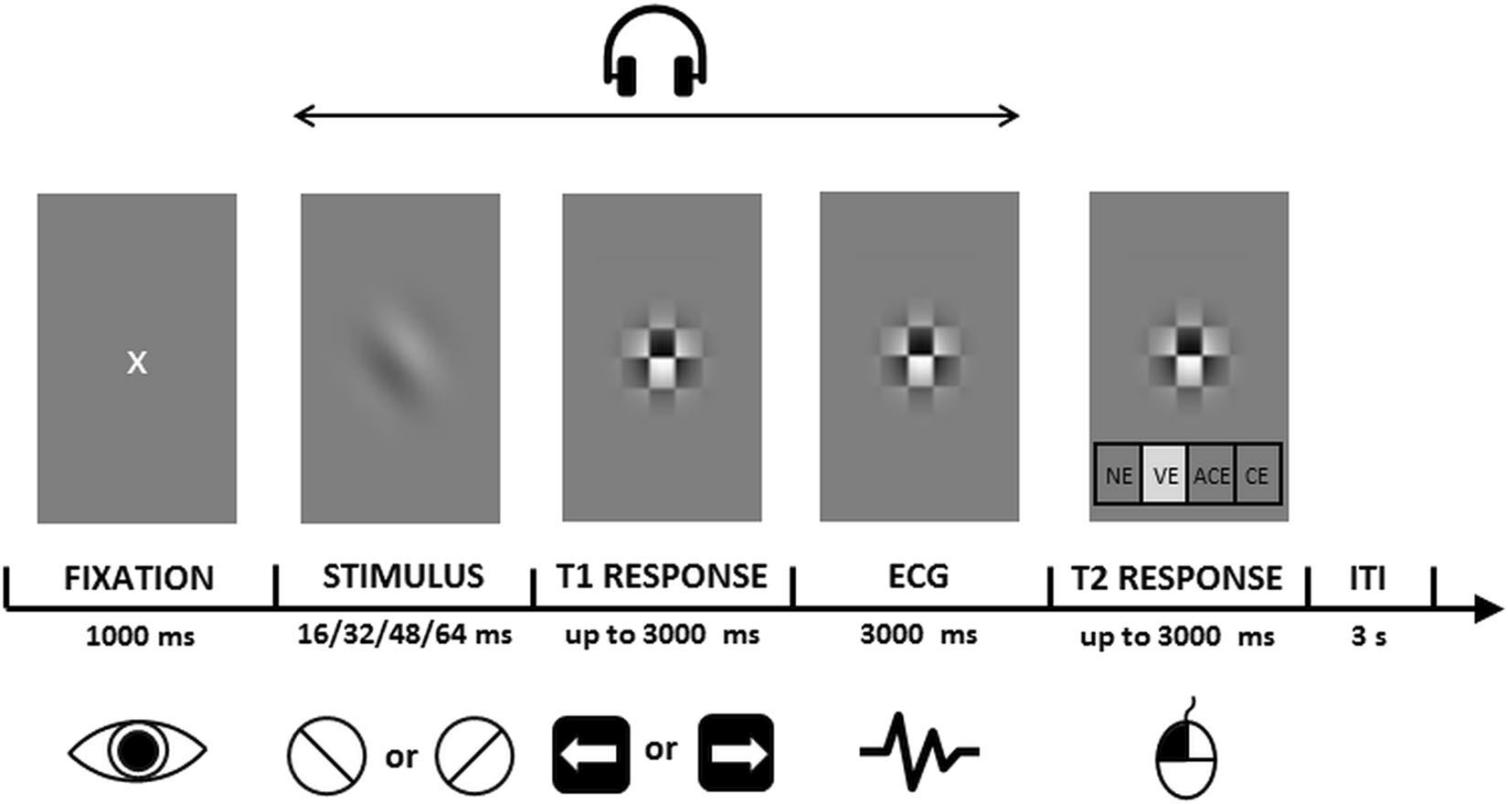
Gabor patch orientation discrimination task. Schematic illustration of a trial flow: each trial started with a fixation cross displayed for a fixed duration of 1000 ms. Then, the target stimulus was presented for 16, 32, 48, or 64 ms and was immediately followed by the mask, which stayed on the screen until the end of the trial. Next, the subject indicated the orientation of the target stimulus by pressing the left or right arrow button on a keyboard. The T1 response-related cardiac activity was subsequently registered using ECG for 3000 ms. Finally, the visibility rating was required. The inter-trial interval lasted 3000 ms. In Experiment 2, real or fake auditory feedback about cardiac activity was delivered through headphones starting with the stimulus presentation and lasting until the Perceptual Awareness Scale^48^ appeared on the screen. In both experiments we measured baseline ECG activity before the stimulus presentation.

Participants underwent an orientation discrimination backward masking task with Gabor patches displayed with constant contrast but varying presentation times (see: Fig. 1). They had to identify Gabor patch orientation (Type 1 decision) and then judge subjective visibility of a stimulus (Type 2 decision) with the Perceptual Awareness Scale^48^. Using ECG, we recorded cardiac activity in the 3-second window between Type 1 and visibility rating responses. In Experiment 2, we additionally manipulated access to information about cardiac activity by delivering auditory information about heartbeats^45^. We employed the same task as in Experiment 1, but with additional auditory cardiac feedback in two conditions: real and fake. Detailed information concerning stimuli parameters, timing, number of trials, ECG recording, and cardiac feedback manipulation is presented in the Method section and Supplementary materials.

### Experiment 1

**Figure 2:**
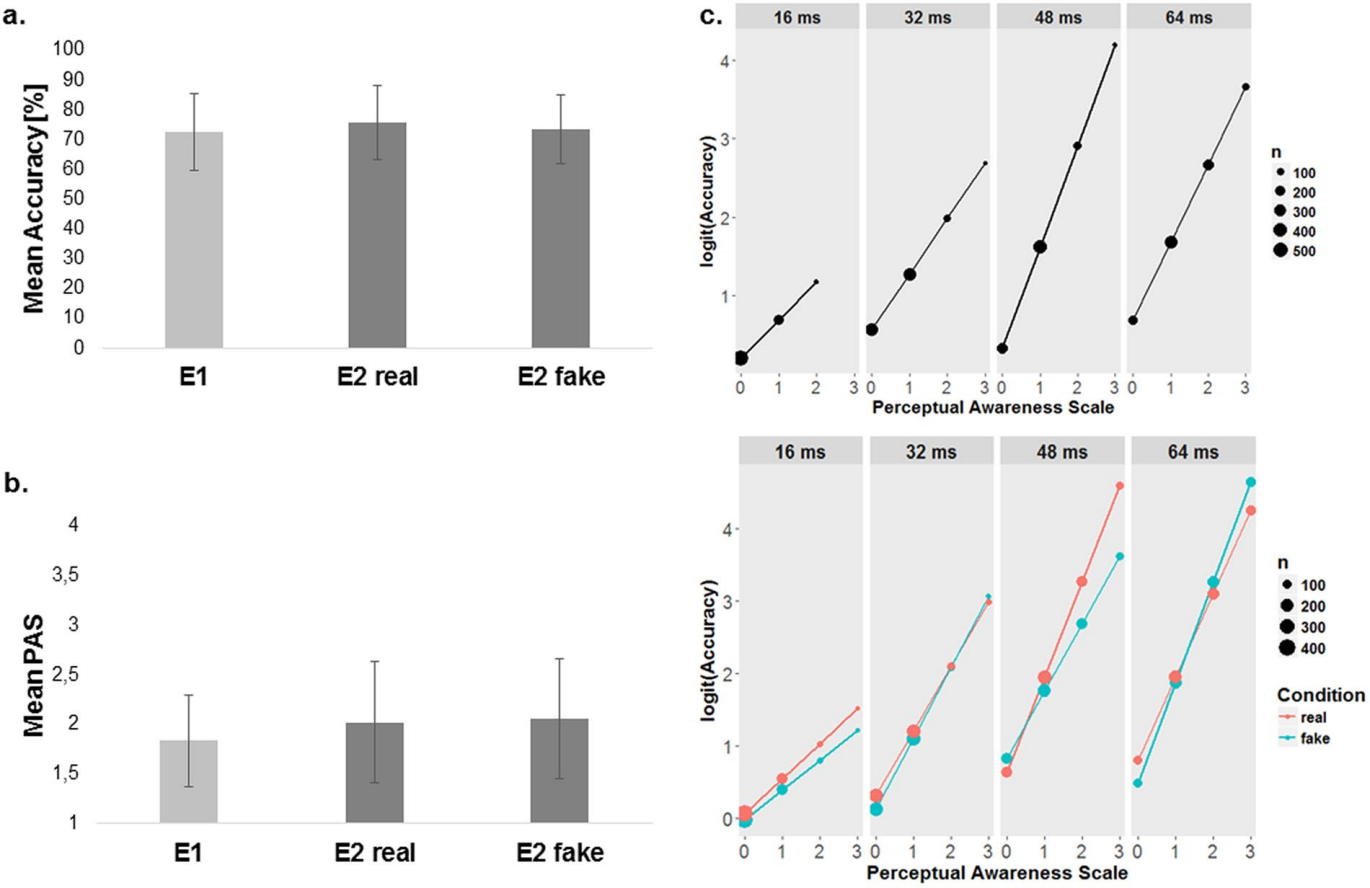
Overview of behavioural results. (**a**) Mean accuracy in the Gabor patch orientation discrimination task was around 75%; it did not differ between Conditions in Experiment 2 and was similar in Experiment 1. (**b**) Mean visibility was around 2 (VE, vague experience); it did not differ between conditions in Experiment 2 and was similar in Experiment 1. (**c**) The results of logistic mixed regression analysis revealed that in both experiments (upper panel: Experiment 1, lower panel: Experiment 2) PAS ratings predicted Gabor patch orientation discrimination accuracy at each Presentation time (columns). However, in Experiment 2, there was no significant difference between real and fake Conditions in metacognitive accuracy (lower panel). PAS predicted accuracy the same way for both Conditions (the slopes do not differ significantly). In (**a** and **b**) the error bars represent standard deviation. In (**c**) X-axis values correspond with the following PAS ratings: 0 - No experience, 1 - Vague experience, 2 - Almost clear experience, and 3 - Clear experience.

Average Gabor discrimination accuracy was 73% (±13%, range 52–94%; see: Fig. 2a) and the average PAS rating equalled 1.83 (±0.46, range 1.13–2.98; see: Fig. 2b).

#### Metacognitive accuracy

The mixed logistic regression analysis revealed that accuracy for the lowest Visibility rating (NE) differed significantly from the chance level only for the 32 and 64 ms Presentation times (see: Fig. 2c – upper panel and Supplementary Table S1; for individual differences in metacognitive accuracy – see Supplementary Figure S1). For all Presentation times, the Visibility rating significantly predicted accuracy level.

#### Cardiac activity

**Figure 3:**
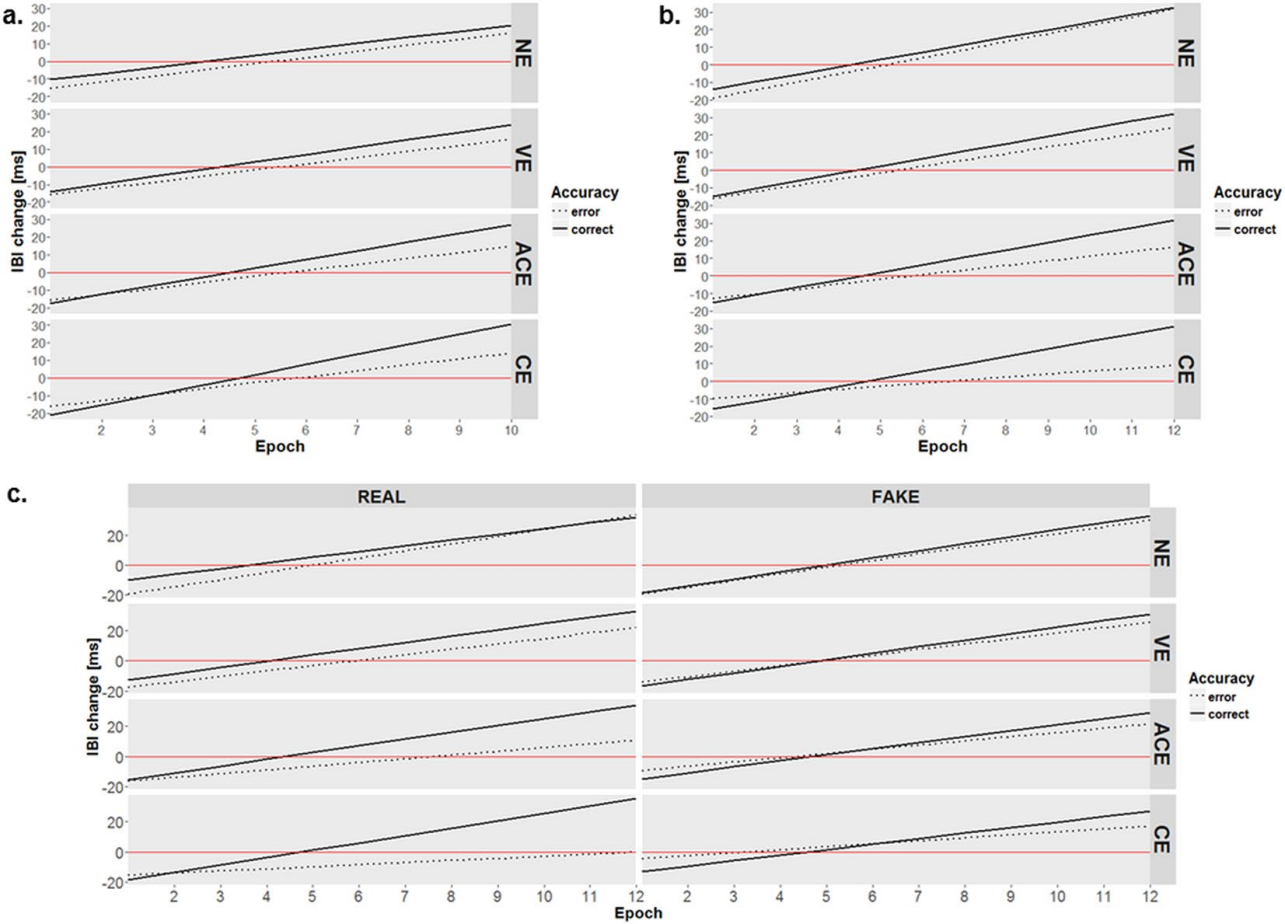
Cardiac activity. Results of the linear regression mixed model analyses comparing cardiac activity following correct versus incorrect stimuli discrimination. All plots depict changes predicted by the model in duration of inter-beat intervals in the 3 seconds between stimulus discrimination (Type 1) and presentation of the Perceptual Awareness Scale (Type 2). All IBIs were pre-stimulus baseline-corrected and averaged in Epochs ((**a**) ten 300 ms Epochs in Experiment 1, and (**b**) twelve 250 ms Epochs in Experiment 2). Predicted IBI change values below the red line mean that the heart was decelerating, whereas above means accelerating. Therefore, the heart rate decelerated just after the Type 1 response in both Experiments (**a)** and (**b**) and then accelerated. However, dynamics of cardiac activity differ depending on the stimuli discrimination accuracy: the heart accelerated slower after incorrect (dotted lines) than correct (solid lines) Type 1 responses. We interpret this difference as cardiac information about Type 1 accuracy. Interestingly, the difference in the cardiac activity following correct versus incorrect stimuli discrimination increased with increasing visibility (PAS rating are depicted in rows). (**c**) In Experiment 2, the cardiac information about Type 1 accuracy was found only in the real cardiac feedback condition. Fake cardiac feedback disrupted the accuracy-dependent pattern of cardiac activity.

The mixed model regression analysis with Epoch (10 levels: [0–300 ms]–[2700–3000 ms]), Visibility rating (4 levels: NE, VE, ACE, CE), Accuracy (2 levels: error, correct), their interaction as fixed effects, and subject-specific random intercept and Visibility rating slope revealed that in incorrect trials when participants did not see the stimuli (i.e. used the lowest Visibility rating – “No experience”), the inter-beat interval change (see: Method for details) over the first 300 ms following T1 response was significantly different from 0 (*z* = −3.54, *p* < 0.001; see: Fig. 3 and Table 1 – left panel, first row – Intercept). The negative value means that compared to the pre-stimulus baseline, the heart rate was beating slower just after the T1 response. Moreover, we found that after incorrect T1 responses the heart rate accelerated by approximately 3.48 ms every 300 ms (*z* = 10.88, *p* < 0.001; see: Table 1 – left panel, row 2). There was no evidence for any Visibility-dependent difference in IBI change in the first 300 ms following incorrect T1 responses (*z* = −0.11, *p* = 0.911; see: Table 1 – left panel, row 3). When participants reported seeing nothing (i.e. at the lowest Visibility rating), the heart rate in the first 300 ms was beating significantly faster after correct T1 responses than after incorrect ones (*z* = 2.27, *p* = 0.023; see: Table 1 – left panel, row 4). There was no evidence for any Visibility-dependent difference in the dynamic of cardiac activity following incorrect T1 responses (*z* = −0.04, *p* = 0.929; see: Table 1 – left panel, row 5). We did not find evidence for any Accuracy-dependent difference in the dynamic of cardiac activity following T1 responses at the lowest Visibility rating (*z* = −0.06, *p* = 0.886; see: Table 1 – left panel, row 6). There was also no evidence for any Visibility-dependent difference in IBI change in the first 300 ms following T1 responses (*z* = −1.28, *p* = 0.2; see: Table 1 – left panel, row 7). Finally, and most importantly, the analyses revealed a non-significant trend in the predicted direction; this suggests that the Accuracy-dependent difference in the dynamics of cardiac activity following T1 responses increases with increasing Visibility rating (*z* = 1.76, *p* = 0.078; see: Table 1 – left panel, row 8).

**Table 1:** Regression coefficients for the regression mixed model for IBI change in both experiments.

	Experiment 1					Experiment 2				
	Estimate	SE	*z*	*p*		Estimate	SE	*z*	*p*	
Intercept	−15.31	4.33	−3.54	0.001	***	−19.13	4.57	−4.18	0.001	***
Epoch	3.48	0.32	10.88	0.001	***	4.59	0.23	20.39	0.001	***
PAS	−0.34	3.07	−0.11	0.911		3.10	2.1	1.47	0.141	
Accuracy	4.93	2.17	2.27	0.023	*	5.03	1.85	2.74	0.006	**
Epoch: PAS	−0.04	0.41	−0.09	0.929		−0.96	0.25	−3.77	0.001	***
Epoch: Accuracy	−0.06	0.40	−0.14	0.886		−0.36	0.28	−1.26	0.208	
PAS: Accuracy	−3.19	2.49	−1.28	0.200		−3.67	1.84	−2	0.045	*
Epoch: PAS: Accuracy	0.80	0.46	1.76	0.078	.	0.97	0.28	3.51	0.001	***

Left panel represents intercept and slopes for Experiment 1, right panel for Experiment 2. Experiment 1: N = 26 # observations = 31829. Experiment 2: N = 27 # observations = 59271.

### Experiment 2

Average Gabor discrimination accuracy (see: Fig. 2a) did not significantly differ between Conditions (*t*_*(26)*_ = 1.76, *p* = 0.09 two-sided, mean difference = 2.2%, 95% CI [5.62%, 8.31%]) and equalled 75% (±12%, range 50–95%) and 73% (±12%, range 51–91%) in the real and the fake cardiac feedback Conditions, respectively. Similarly, there was no difference in average PAS rating (*t*_*(26)*_ < 1, *p* > 0.9 two-sided) between real (mean = 2.02 ± 0.62, range 1.04–3.4) and fake (mean = 2.05 ± 0.61, range 1.08–3.43) Conditions (see: Fig. 2b).

#### Metacognitive accuracy

The mixed logistic regression analysis did not exhibit any significant differences between Conditions (see: Fig. 2c – lower panel and Supplementary Table S2; for individual differences in metacognitive accuracy – see Supplementary Figure S1). Namely, neither accuracy at the lowest rating (intercepts), nor metacognitive accuracy (slopes) differs for any Presentation time between real and fake Conditions. However, a non-significant trend in the predicted direction which suggests a difference between Conditions (*z* = −1.76, *p* = 0.078) in metacognitive accuracy was found for 48 ms presentation time; this in turn suggests that Visibility ratings predicted accuracy better in the real feedback Condition.

#### Cardiac activity

In order to check whether the pattern of results observed in Experiment 1 was replicated in Experiment 2, we fitted the same model as in Experiment 1 before comparing the T1 response-related cardiac activity between Conditions. We found the same (and even stronger) effects as in Experiment 1 (see: Fig. 3b and Table 1 – right panel): significant Accuracy-dependent differences in heart rate deceleration in the first 300 ms following T1 response (*z* = 2.74, *p* = 0.006; Table 1 – right panel) 4. row) and a significant increase in Accuracy-dependent differences in the dynamic of cardiac activity following T1 response with increasing Visibility rating (*z* = 0.97, *p* < 0.001; Table 1 – right panel, 8. row). Therefore, we replicated the results from Experiment 1 which suggest that that there is information about discrimination accuracy coded in T1-response-related cardiac activity. Additionally, we found a Visibility-dependent difference in the dynamic of cardiac activity following incorrect T1 responses: heart rate acceleration within 3000 ms following incorrect T1 responses was less pronounced with increasing Visibility ratings (Table 1 – right panel, 5. row, *z* = −3.77, *p* < *0.001*). Moreover, we found that the Accuracy-dependent difference in IBI change in the first 300 ms following T1 responses kept increasing with increasing visibility judgement (Table 1 – right panel, 7. row, *z* = −3.67, *p* = 0.045). When participants reported seeing nothing, in the first 300 ms following T1 response the heart was beating slower after incorrect as compared to correct T1 responses, and the pattern reversed when they saw a stimulus clearly.

Next, we fitted two models (for details see: Method) to compare coefficients between real and fake cardiac feedback Conditions (see: Fig. 3c and Table 2 – right panel) and test effects within Conditions analogically to the tests described above (see: Table 2 – left panel: real Condition, Table 2 – middle panel: fake Condition). Analyses revealed that Accuracy-dependent cardiac activity differs between Conditions (*z* = −8.65, *p* = 0.019; Table 2 – right panel, row 4): when participants saw nothing, the heart rate was beating slower in the first 300 ms after incorrect than after correct T1 responses only in the real cardiac feedback Condition (real: *z* = 9.33, *p* < 0.001; Table 2 – left panel, row 4; fake: *z* = 0.68, *p* = 0.26; Table 2 – middle panel, row 4). Moreover, in trials in which participants reported seeing nothing, the Accuracy-dependent difference in the dynamic of cardiac activity differed between Conditions (*z* = 1.09, *p* = 0.035; Table 2 – right panel, row 6): in the real Condition, the heart accelerated more profoundly after incorrect than after correct trials (*z* = −0.96, *p* = 0.018; Table 2 – left panel, row 6). However, in the fake condition there was no such difference (*z* = 0.24, *p* = 0.547; Table 2 – middle panel, row 6). Finally, there was a non-significant trend in the predicted direction; this suggests that Accuracy-dependent difference in the dynamic of cardiac activity dissimilarly changes with increasing Visibility rating in the real compared to the fake Condition (*z* = −1.02, *p* = 0.067; Table 2 – right panel, last row). The models fitted within Conditions revealed that only in the real feedback Condition did the Accuracy-dependent difference in the dynamic of cardiac activity increase with increasing Visibility rating (*z* = 1.49, *p < *0.001*;* Table 2 – left panel, last row). Specifically, we observed that the more pronounced the difference between cardiac response following correct versus incorrect stimuli discrimination (T1 response), the better participants saw the stimuli, but only when they were listening to their own heartbeats, not someone else’s.

**Table 2:** Between Conditions comparison of regression coefficients for the regression mixed model for IBI change in Experiment 2.

	Real cardiac feedback					Fake cardiac feedback					Condition comparison				
	Estimate	SE	*z*	*p*		Estimate	SE	*z*	*p*		Estimate	SE	*z*	*p*	
Intercept	−19.14	4.83	−3.96	0.001	***	−19.17	4.79	−4.00	0.001	***	−0.03	2.93	−0.01	0.991	
Epoch	4.76	0.33	14.58	0.001	***	4.45	0.31	14.28	0.001	***	−0.31	0.45	−0.69	0.490	
PAS	1.23	2.74	0.45	0.652		4.79	2.63	1.82	0.068	.	3.55	3.31	1.07	0.284	
Accuracy	9.33	2.64	3.53	0.001	***	0.68	2.57	0.26	0.792		−8.65	3.68	−2.35	0.019	*
Epoch: PAS	−1.13	0.37	−3.05	0.002	**	−0.81	0.35	−2.30	0.021	*	0.32	0.51	0.63	0.532	
Epoch: Accuracy	−0.96	0.41	−2.36	0.018	*	0.24	0.40	0.60	0.547		1.19	0.57	2.11	0.035	*
PAS: Accuracy	−4.10	2.63	−1.56	0.119		−3.01	2.52	−1.19	0.232		1.09	3.61	0.30	0.762	
Epoch: PAS: Accuracy	1.49	0.40	3.72	0.001	***	0.47	0.38	1.22	0.221		−1.02	0.56	−1.83	0.067	.

Left panel represents intercept and slopes for the real cardiac feedback condition, middle panel for the fake cardiac feedback, and right for comparison of between Conditions coefficients. N = 27 # observations = 59271. p < 0.1, *p < 0.05; **p < 0.01; ***p < 0.001.

## Discussion

Our results demonstrate that there is interoceptive information about accuracy (internal feedback) that may serve as evidence when judging subjective visibility. Moreover, we showed that delivering fake auditory feedback about cardiac activity disrupts cardiac information about accuracy. However, we failed to demonstrate a direct impact of cardiac information about stimuli discrimination accuracy on metacognitive accuracy.

Firstly, we demonstrated that the heart accelerates less after incorrect as compared to correct stimulus discrimination (Type 1 perceptual decision). This result is in line with previous studies describing accuracy-dependent cardiac activity^31,41,43^. The observed difference may be interpreted as an effect of phasic activity in the locus coeruleus–norepinephrine (LC-NE) system^49^ following detection of salient internal stimuli, such as an error^33^. It was also suggested that motivationally significant events trigger phasic LC-NE activity in order to increase neurons’ responsivity to afferent input in regions receiving projection from the LC-NE system^50^, thus they can facilitate behavioural response to an event. Moreover, P3 is recognized as an electrophysiological correlate of phasic LC-NE activity^51^ evoked by salient external stimuli. Importantly, an analogue interpretation was proposed for the error-related Pe component, suggesting that it reflects a P3-like facilitation of neuronal responsivity following salient internal stimuli, such as an error^33,52^. Moreover, it was suggested that LC-NE activity triggered by a motivationally significant event also influences the autonomic nervous system^53^. It has been suggested that error-related changes in interoceptive states (such as heart rate deceleration, pupil dilation, skin-conductance response) are evoked by phasic activity of the LC-NE system^33,54^. Studies on rats support this assumption by demonstrating LC-NE influences on cardiac activity^55–58^. Moreover, the AIC has been argued to be anatomically connected to the LC^59,60^ and insula cortex stimulation elicits cardiovascular changes^61^. Therefore, due to the identified direct and indirect (via the anterior cingulate cortex – ACC^62,63^) connections between the AIC and the LC, it is possible that AIC activity might impact phasic LC activity and elicit cardiovascular changes. Thus, we hypothesize that the smaller acceleration following an incorrect rather than a correct Type 1 decision observed in our study may be cautiously interpreted as an indicator of an orienting response toward an error as a motivationally salient stimulus. Nonetheless, further neuroimaging studies are needed to verify this hypothesis and investigate the relation between error detection, salience processing, AIC activity, LC-NE system phasic activity, and heart rate variability.

Secondly, we found that accuracy-dependent differences in the dynamic of cardiac response following Type 1 decisions increase with visibility. Namely, the greater the difference between heart activity following erroneous and correct stimulus discrimination response, the more visible the stimulus was judged to be. A question remains about the causal direction of this relation, which is clearly important for the interpretation of the observed pattern of the results. On one hand, if we interpret the accuracy-dependent difference in the dynamic of Type 1 response-related cardiac activity as an indicator of the amount of interoceptive information about Type 1 accuracy fed to a Type 2 decision, we may conclude that the amount of accuracy-specific internal feedback information coded in cardiac activity influences subjective visibility. This conclusion would be in line with models that suggest that metacognitive accuracy depends on some additional post Type 1 decision processing^35^. However, the results of Experiment 2 show that disruption of the accuracy-dependent pattern of cardiac activity does not influence general metacognitive accuracy. Thus, an alternative interpretation may be that the more salient the stimulus is, the greater the cardiac change it evokes and thus the observed pattern of cardiac activity is stimulus-evoked rather than response-evoked. On the other hand, this interpretation cannot in turn explain why increasing visibility enhances the increasing difference in cardiac activity following a correct versus incorrect Type 1 decision. Thus, disentangling the causal direction requires further investigation with better control for stimulus- and response-evoked effects on cardiac activity.

Finally, in Experiment 2 we demonstrated that delivering fake auditory feedback about heart rate disrupts the accuracy-dependent pattern of cardiac activity described above. When participants listened to someone else’s heartbeats, their cardiac activity following stimulus discrimination did not differ depending on discrimination accuracy. Thus, it seems that accuracy-dependent cardiac information was lost when we introduced misleading auditory feedback about cardiac activity. Importantly, we did not find any difference between real and fake cardiac feedback conditions in the means of Type 1 accuracy, PAS rating, or metacognitive accuracy (i.e. correspondence between PAS rating and accuracy). Therefore, it seems that cardiac information about Type 1 response accuracy influences neither general visibility judgement nor averaged metacognitive accuracy, but rather the amount of information fed from erroneous responses to visibility judgments. However, there are few issues to be resolved before drawing the aforementioned conclusion. First, we need to consider how the brain processes real and fake cardiac feedback and how it can further affect cardiac activity. Recently, N1 (early visual evoked potential in EEG) suppression was observed when participants listened to heartbeat-related sounds, which suggests that the brain processes cardiac feedback similarly to other self-generated sounds^64^. Another recent study demonstrated that people are able to distinguish between pre-recorded sounds of their own and someone else’s heartbeats^65^, but only if the sound is recorded using a Doppler device. Our study also supports this result: we delivered cardiac feedback in the form of sounds triggered by real-time detected QRS complexes in an ECG signal. We found that only 9 of 27 participants noticed a difference between real and fake cardiac feedback (see: Supplementary Table S5); interestingly, a comparable number of participants in both conditions (real: 22 *vs* fake: 19) agreed that they were listening the sound of their own heartbeats. Therefore, in our study people tended to experience the presented cardiac feedback as if it was real.

Secondly, fake feedback could disrupt accuracy-dependent cardiac activity by entrainment of a cardiac rhythm to the sound^66,67^. Namely, listening to the rhythmical sounds of fake cardiac feedback may change a participant’s heart rate. Moreover, taking into consideration that the majority of participants experienced the fake cardiac feedback as if it was real, we hypothesise that it actually changes participants’ predictions about their own heartbeat: they expected that their own heartbeat would be coherent with the fake rhythm. Thus, in the context of the interoceptive predictive coding approach^68–70^, it may be that participants’ heartbeats synchronized with the fake one to minimize prediction error. Thus, listening to someone else’s heartbeat might possibly disrupt the accuracy-dependent cardiac activity pattern. However, since we did not monitor the exact timing of the particular fake heartbeats, we cannot assess how exactly it influences participants’ cardiac activity at the level of a single heartbeat. Thus, before concluding that listening to fake auditory feedback about cardiac activity has no direct impact on metacognitive accuracy even though it disrupts the accuracy-dependent cardiac activity pattern, we need to better understand how listening to someone else’s heartbeats affects ongoing cardiac activity. Further investigation is needed with fake feedback timing controlled on a beat-by-beat basis and monitoring of beat-to-beat changes.

In the present study, we demonstrated an accuracy-dependent pattern of results in cardiac activity. Firstly, we observed that the heart accelerated less after incorrect compared to correct stimuli discrimination. We interpret this difference as a marker of the orienting response toward an error: internal motivationally significant stimuli. Secondly, we have shown that the difference in the dynamic of the heartbeat change following T1 response between correct and incorrect trials is clearly related to visibility judgments; therefore, the difference in the change is more pronounced in the visible than the invisible trials. We propose that cardiac information about T1 response accuracy is fed to visibility ratings. Finally, we observed the aforementioned pattern only when participants listened to their own but not someone else’s heartbeats. However, we did not find any direct difference in metacognitive accuracy between real and fake cardiac feedback conditions.

## Method

### Participants

Twenty-nine participants took part in Experiment 1 (16 females) and 33 in Experiment 2 (22 females). Three participants in Experiment 1 and six participants in Experiment 2 were excluded from further analysis (for details - see: Supplementary materials). Therefore, in total data from 26 (14 females; 23.1 ± 4.7 years, range 17–40 years) and 27 (19 females; 24.3 ± 4.6 years, range 18–41 years) participants was analysed in Experiment 1 and Experiment 2, respectively. Since it was the first experiment of this type, sample size was estimated on the basis of sample sizes in a backward masking task in our lab (N between 20 and 30) and on the basis of a previous experiment concerning error-related cardiac changes^31^ (N = 22). All participants gave written informed consent to participation in the study and were paid 20 PLN. The experimental protocol was approved by the Committee for Research Ethics of the Institute of Psychology of Jagiellonian University (decision 26/04/2016) and adhered to the tenets of the Declaration of Helsinki.

### Stimuli and apparatus

The target stimulus was a Gabor patch (sinusoidal gratings; size: 2 degrees of visual angle; sigma of the Gaussian envelope = 30 pixels) at a spatial frequency of 20 cpd, tilted 45° to the right or left of vertical. It was presented in the centre of a screen on a light grey background (rgb(128, 128, 128)) with constant contrast (30%). The backward mask was a Gaussian vignetted checkerboard with the same parameters as the Gabor patch.

Subjective visibility was measured by means of the Perceptual Awareness Scale^48^ presented in the lower part of the screen. The PAS was developed on the basis on the phenomenology of visual experience and is used to probe the subjective experience of seeing in visual psychophysical tasks. Participants rate each stimulus visibility by choosing one of the four possible ratings: no experience (NE), vague experience (VE), almost clear experience (ACE), clear experience (CE). In our task, the four possible ratings were ordered along a horizontal plane, with each presented in a separate rectangular frame; the chosen ratings were highlighted.

All stimuli were displayed via a custom Python script on a Benq XL2411-B monitor (resolution: 1920 × 1080 pxl; refresh rate: 100 Hz) in Experiment 1 and an LG 22EN33 monitor (resolution: 1920 × 1080 pxl; refresh rate: 60 Hz) in Experiment 2. Participants indicated Gabor orientation (T1 response) by pressing the arrow keys on a keyboard with their left hand and rated visibility (T2 response) using a mouse with their right hand.

### Electrocardiography (ECG)

In Experiment 1, heart rate was recorded from 43 mm diameter Ag/AgCl ECG foam electrodes filled with a solid gel attached to the chest in a triangle and connected to an S&W/BIAZET ECG monitor (sampling rate 1000 Hz). The output from an R-wave peak detector was used to compute R–R intervals (inter-beat intervals, IBI) in ms. Inter-beat interval durations were averaged for each subject in each trial and segmented into epochs over the interval between T1 response and PAS presentation. Given that the break had a constant duration of 3000 ms (see below), averaged IBI values were computed for 10 epochs, each lasting 300 ms. Additionally, baseline IBI value from 300 ms preceding the target stimulus presentation^31^ was computed for each person in each trial.

In Experiment 2, we recorded heart rate by means of an Arduino™ microcontroller with a mounted e-Health Sensor Shield v2.0 from Libelium™ combined with three electrodes placed on the chest (sampling rate was 84 Hz). The raw signal was real-time analysed to detect QRS complexes using a custom implementation of the Pan-Tompkins algorithm^71^ (for details – see: Supplementary materials). We averaged the signal in 12 epochs, each lasting 250 ms (in total 3000 ms between T1 response and PAS appearance). Baseline was an averaged IBI from 250 ms preceding target presentation in a given trial.

### Cardiac feedback

In Experiment 2, we delivered auditory cardiac feedback in two possible Conditions: real and fake. The feedback was played starting after the target stimulus presentation until the PAS appeared on the screen. In the real cardiac feedback Condition, every time an R-peak was detected by ECG, a bass sound resembling the sound of a beating heart during systole was triggered. In the fake cardiac feedback Condition, we delivered pre-recorded auditory heartbeat patterns adjusted to an average participant’s heart rate (for details see: Supplementary materials).

### Procedure

After attaching ECG electrodes, participants were seated at an approximately 60 cm viewing distance. First, as a part of a separate project, individual differences in interoceptive abilities (all the three dimensions proposed by Garfinkel and colleagues^72^: accuracy, sensibility, and awareness) were measured by means of Schandry’s^73^ heartbeat tracking task and Awareness Scale from the Porges’ Body Perception Questionnaire^74^ (for details – see: Supplementary materials). Then, participants underwent two training sessions of the orientation discrimination backward masking task, each consisting of 10 trials (for details – see: Supplementary materials). In the experimental session, at the beginning of each trial a fixation cross (shape: X; rgb(255,255,255)) was displayed for a fixed duration of 1000 ms. The target stimulus was then presented for 16, 32, 48, or 64 ms and was immediately followed by the mask, which stayed on the screen until the end of the trial. Participants indicated the orientation of the target stimulus by pressing the left or right arrow key. We introduced a 3000 ms break between T1 response and PAS presentation in order to record T1-response-related cardiac activity. During this period, nothing changed on the screen and participants were asked to sit still until the scale appeared; they were informed that during this period their cardiac activity would be recorded. After they rated visibility, the screen froze for a 3000 ms inter-trial interval. Each presentation time was repeated 32 times; in total, the experiment consisted of 128 trials and was administered in 4 blocks with short breaks between each. In each block, trials were presented in a randomized full factorial design order. No feedback in the experimental session was delivered.

The procedure in Experiment 2 was similar to Experiment 1 with a few exceptions: (1) real or fake cardiac feedback was played; (2) trials were blocked with respect to the two cardiac feedback Conditions. Each participant underwent 6 blocks consisting of 32 trials: 3 with the real and 3 with the fake cardiac feedback (in total 96 trials per Condition). The order of the Conditions was counterbalanced between participants. We kept participants naïve about the two types of cardiac feedback.

### Statistical analyses

In both experiments, we excluded all trials with missing T1 (E1: on average 3.25 ± 3.73% of all trials, range 0–13.28%; E2: on average 3.32 ± 6.06% of all trials, range 0–23.44%) and/or T2 response (E1: on average 1.14 ± 2.27% of all trials, range 0–9.38%; E2: on average 0.15 ± 0.43% of all trials, range 0–2.08%). We use the ± symbol for standard deviation. For all analyses, significance level alpha equals 0.05. Since the Shapiro-Wilk tests did not reveal a violation of normality (for accuracy: *W* = 0.953, *p* = 0.254; for PAS rating: *W* = 0.986, *p* = 0.965), we conducted paired-sampled t tests to compare average accuracy and average PAS (i.e. Visibility) rating between the real and fake Conditions.

### Metacognitive accuracy

Metacognitive accuracy was operationalized as the relationship between the accuracy of Gabor patch orientation discrimination (i.e. Type 1 response) and the visibility rating (Type 2 response)^22,75^. The relation between visibility and accuracy was analysed using logistic regression, which is the correct model for predicting binary outcomes such as accuracy. Therefore, the mixed logistic regression models were fitted using the lme4 package^76^ in the R Statistical Environment^77^ using standard (0/1) contrast coding.

In Experiment 1, we fitted a model separately for each Presentation Time (4 levels: 16, 32, 48, 64 ms), with the Visibility rating (4 levels: NE, VE, ACE, CE) as the fixed effect and subject-specific random intercept. Visibility ratings were centred on the lowest values (NE). Therefore, the intercept informs about performance level when participants report seeing nothing, and the regression slope reflects metacognitive accuracy, i.e. the relation between visibility (Type 2) and accuracy (Type 1) (for a detailed explanation see Supplementary materials). In Experiment 2, we ran the same analysis with one exception: we additionally introduced to the model Condition (2 levels: real, fake) and its interaction with Visibility ratings as fixed effects and we fitted within-Conditions models.

### Cardiac activity

The raw IBI data was inspected to detect any potential artefacts in recordings. Then, data was baseline corrected (for details of ECG signal pre-processing – see: Supplementary materials).

In Experiment 1, we analysed differences in IBI change by linear mixed regression (using the lme4 package^76^ for R Statistics^77^) with Epoch (10 levels: [0–300 ms]–[2700–3000 ms]), Visibility rating (4 levels: NE, VE, ACE, CE), Accuracy (2 levels: error, correct), their interactions as fixed effects, and subject-specific random intercept and Visibility rating slope. Visibility rating was centred on the lowest value (NE). Therefore, intercept reflects the average IBI change in the 300 milliseconds following T1 response in incorrect trials with no visual experience (i.e. at the lowest Visibility rating). In Experiment 2, we ran the same analysis with two exceptions: (1) factor Epoch had 12 levels (for explanation see: Supplementary materials), and (2) we introduced Condition to the model (2 levels: real vs. fake) and its interaction with other factors as fixed effects. The basic Condition was real cardiac feedback. Therefore, intercept reflects average IBI change in the 250 milliseconds following T1 response in incorrect trials with no visual experience in the real cardiac feedback Condition (for interpretation of coefficients see: Supplementary materials). Additionally, in order to investigate at which Visibility rating we could observe the accuracy-dependent difference in the dynamic of T1 response-related cardiac activity, we fitted linear regression mixed models within Visibility rating for each experiment separately (for detailed results see: Supplementary materials). We decided to use mixed regression analysis due to the multilevel data structure individual differences in heart rate variability (for details see: Supplementary materials), and visibility rating.

Additionally, in order to control for possible reaction time influences, in both experiments we fitted the additional model with RT as a fixed factor (see: Supplementary materials). Since we observed similar coefficients and patterns of results, for the sake of simplicity of result presentation and visualization, we decided to report here the results of the simpler model.

### Data

Data and scripts are available here: osf.io/pq24j.

## Electronic supplementary material

Supplementary materials
