# Supplementary material for: Error-related cardiac response as information for visibility judgements
Source: Sci Rep. 2018 Jan 18;8:1131. doi: 10.1038/s41598-018-19144-0 (PMC5773515; doi:10.1038/s41598-018-19144-0)
Supplement: Supplementary file 1 — Supplementary materials [file 41598_2018_19144_MOESM1_ESM.pdf]

Supplementary materials:
Error-related cardiac response as information for visibility judgements.

Marta Łukowska[1,*], Michał Sznajder[2] & Michał Wierzchoń[3]

[1,2,3] Consciousness Lab, Institute of Psychology, Jagiellonian University, Cracow, 30-060, Poland

[*] mar.lukowska@gmail.com

Supplementary method

Participants' exclusion criteria

In Experiment 1, three participants were excluded from further analysis: one because of a below-chance level performance (i.e. 32%), and another two due to an insufficient number of valid trials (below 75% of all trials) resulting from either omissions in the discrimination task, lack of awareness rating, or problems with the ECG recording. In Experiment 2, six participants were excluded due to misunderstanding of the experimenter's instructions and missing trials (2), accuracy below chance level (< 50 %) (1), or a problem with the ECG recording (3).

Interoceptive abilities

Heartbeat tracking task[1] that probes interoceptive ability: namely, heartbeat tracking accuracy[2]. In the task, participants were asked to close their eyes and detect and count their heartbeats for intervals of 25, 35, 45, or 100 seconds. The beginning and end of each counting interval was signalled with a sound (duration = 200 ms; $pitch_{start}$ = 400 Hz, $pitch_{stop}$ = 500 Hz) via speakers. After the stop signal, in each trial participants were asked 1) how many heartbeats they counted and 2) how confident they were about their response (using a visual analogue scale ranging from 0 to 100 in increments of 1). Each trial was repeated twice, so in total each participant underwent 8 experimental trials delivered in a randomised order. The task was programmed using the ExpyVR software (http://lnco.epfl.ch/expyvr) developed by Javier Bello Ruiz and Bruno Herbelin at the Laboratory of Cognitive Neuroscience (LNCO), EPFL. Additionally, participants filled in a Polish version of the Awareness Scale from the Porges' Body Perception Questionnaire[3]. Importantly, we measured individual differences in interoceptive abilities as a part of a separate project. Therefore, results of these tests are reported elsewhere[4].

Training session

Participants first underwent two training sessions, each consisting of 10 trials. Trial structure was the same as in the experimental session with three exceptions: (1) presentation time was longer (328 and 128 ms in the first and second training session, respectively); (2) trial-by-trial feedback about accuracy was provided; (3) no visibility ratings were required.

Real-time QRS detection and cardiac feedback

The raw signal was analysed in real-time to detect QRS complexes using a custom implementation of the Pan-Tompkins algorithm[5,6]. Signal processing was divided into two stages: filtering and thresholding. Every raw ECG measurement was first filtered using a Butterworth band-pass filter[7] with desired lower cut-off frequency (5 Hz) and upper cut-off frequency (15 Hz). The band-

pass filtered signal was then varied to identify signal segments with high value changes. These changes were then squared and integrated and QRS complexes were marked. In the thresholding stage, previously marked QRS complexes were classified as valid or caused by noise using a set of real-time dynamically set thresholds (based on previously detected QRS and noise peaks in order to adjust to the most recent noise level). The system of dynamic thresholds combined with complex filtering makes it possible to achieve a good level of detection sensitivity with a relatively low level of false positive QRS complex detections.

For the fake cardiac feedback, using an ECG and a real-time QRS detector we recorded the heart activity of six volunteers with average heart rates matching one of the six heart rate categories: 45-55 BPM, 55-65 BPM, 65-75 BPM, 75-85 BPM, 85-95 BPM, 95-105 BPM. We choose the following categories to cover all resting state heart rates of healthy participants that are less physically active (i.e. according to American Heart Association[1] between 60 and 100 bpm), but also those more physically active (i.e. 45 to 65 bpm). We set a step of 10 bpm to keep the fake cardiac feedback within the range of individual heart rate variability and make it indistinguishable from the real cardiac feedback. In order to match the proper category of the fake cardiac feedback, at the beginning of the experiment, we recorded participants' cardiac activity for 1 minute and averaged it. Based on the average, we chose the corresponding fake cardiac feedback category. None of the volunteers took part in the experiments. In both experiments, we used Sennheiser HD 203 headphones to deliver auditory cardiac feedback.

ECG pre-processing

The raw IBI data was inspected to detect any potential artefacts in the recordings. First, we automatically marked all RR intervals differing more than 20% from the previous interval[8]. We then visually inspected all marked intervals to disentangle artefacts from physiological or experimentally evoked heart rate changes. All RR intervals identified as artefacts were excluded from further analysis.

To evaluate heart rate dynamics after T1 response, we computed IBI change for each Epoch in each trial by deviating averaged IBI values in a given Epoch from the pre-stimulus baseline. We decided to use as a baseline the pre-stimulus cardiac activity based on the previous studies on error-related cardiac activity[9] in order to avoid potential differences in the baseline stemming from stimulus-evoked cardiac activity. Therefore, positive and negative IBI change values mean heart rate acceleration and deceleration, respectively. Mixed regression model analysis did not reveal differences in pre-stimulus

[1] Compare: http://www.heart.org/HEARTORG/Conditions/More/MyHeartandStrokeNews/All-About-Heart-Rate-Pulse_UCM_438850_Article.jsp#.WWTIPIjyhEa

baseline depending on Visibility rating, Accuracy, or interaction between them. In Experiment 2, Condition also did not differentiate the pre-stimulus baseline (all *p*s > .05).

We set Epoch duration based on the previous research on stimulus-[10] and response-evoked[9] cardiac activity and taking into account technical restrictions resulting from the output rates of the ECG devices used. Given that in Experiment 1 the R-peak detector output IBI values with a frequency of 10 Hz (i.e. one record every 100 ms), we could not set epoch to 250 ms, so we decided to set it to 300 ms. Therefore, for each Epoch we averaged IBI values from 3 consecutive 100 ms duration records. On the other hand, in Experiment 2 we used another ECG device with a different R-peak detector implementation and an output rate of 84 Hz (i.e. one record every 11.9048 ms). Therefore, in Experiment 2 the output rate allowed us to set epoch duration to 250 ms; for each epoch we averaged IBI values from 21 consecutive records.

Before fitting the linear model during the data exploration phase, we aggregated IBI change and plotted it as a function of Epoch, Accuracy, Visibility rating, and Condition (only in Experiment 2; see: Supplementary Figure S6). After visual inspection, we concluded that the dynamic of cardiac activity in time (i.e. as a function of Epoch) resembles linear. Namely, after primary deceleration (defined in reference to the pre-stimulus baseline) we observed linear heart rate acceleration. Additionally, a Pearson product-moment correlation coefficient was computed to assess the relationship between the IBI change and Epoch and test whether the relation is linear. There was a significant correlation between the two variables in both experiments – Experiment 1: $r = .66$, $p < .001$; and Experiment 2: $r = .87$, $p < .001$.

Supplementary results

Reaction time

In both experiments, we measured Type 1 response reaction time. The average reaction time equalled 776.2 ms (± 569.31 ms) in Experiment 1 and 1055 ms (± 569.31 ms) in Experiment 2. Additionally, in order to compare reaction time between real and fake cardiac feedback Condition, we fitted a linear regression mixed model with Condition (2 levels: real, fake) as fixed effect and subject-specific random intercept. We decided to apply a mixed modelling approach in order to consider high inter- and intra-individual differences in reaction time. We found that RT was significantly slower in the real as compared to the fake cardiac feedback Condition (z = -3.12; p = .002).

Heart rate variability

We found large between- and within-subject variability in duration of inter-beat intervals (see: Supplementary Figure S4). In Experiment 1, group average IBI equals .801 s (± .138 s, range .605-1.176 s) and group average of within-subject standard deviation of IBI is .115 s (± .052 s, range .053-.237 s). In Experiment 2, group average IBI equals .836 s (± .145 s, range .61-1.09 s) in the real cardiac feedback condition and .833 s (± .145 s, range .606-1.434 s) in the fake cardiac feedback condition. Within-subject standard deviation of IBI is .065 s (± .033 s, range .03- .178 s) in the real cardiac feedback condition and .062 s (± .031 s, range .027-.161 s) in the fake cardiac feedback condition. Since the Shapiro-Wilk test did not reveal violation of normality (W = .981, p = .897), we conducted a paired-sampled t test to compare average IBI between the real and fake conditions. The test revealed that average IBI did not differ depending on condition ($t_{(26)}$ = .776, p = .445 two-sided, mean difference = .003 s, 95% CI [-.006 s, .012 s]; see: Supplementary Figure S5). Thus, the observed differences between conditions in the accuracy-dependent pattern of cardiac activity are not due to changes in heart rate variability.

Cardiac activity – within-Visibility-rating model

In order to investigate at which Visibility rating we could observe the accuracy-dependent difference in dynamic of T1-response-related cardiac activity, we fitted additional linear regression mixed models within Visibility rating for each experiment separately (for detailed results see: Supplementary Table S3). In Experiment 1, the model fitted within Visibility rating with Epoch (10 levels: [0-300 ms]-[2700-3000 ms]), Accuracy (2 levels: error, correct), their interaction as fixed effects, and subject specific random intercept did not reveal significant accuracy-dependent difference in the dynamic of cardiac activity following T1 response. However, for the Visibility rating "Almost clear experience" we found a non-significant trend in the predicted direction (z = 1.933, p = .053). In Experiment 2, the same model revealed a significant accuracy-dependent difference in the dynamic of

cardiac activity following T1 response at the two highest Visibility ratings, i.e. when participants reported “Almost clear experience” (z = 2.319, p = .02) and “Clear experience” (z = 2.307, p = .021). We did not find this difference at the two lowest Visibility ratings (i.e. “No experience” and “Vague experience”).

Cardiac activity - analyses with reaction time

In order to control for possible reaction time influences, in both experiments we fitted the additional models with RT as a fixed factor (without interactions). For the models with Epoch, Accuracy, and PAS rating as fixed factors, the inclusion of the reaction time improved model fit in Experiment 2 ($\chi^2_{(1)}$ = 62.399, p = 2.805e-15; see: Supplementary Table S4 – right panel), but not in Experiment 1 ($\chi^2_{(1)}$ = .037, p = .848; see: Supplementary Table S4 – left panel). In Experiment 2, for the model with condition as an additional fixed factor, the inclusion of the reaction time improved model fit ($\chi^2_{(1)}$ = 58.362, p = 2.18e-14; see: Supplementary Table S5 – right panel). Model fit improvement after RT inclusion was also found for the model fitted within conditions ($\chi^2_{(2)}$ = 69.526, p = 7.992e-16; see: Supplementary Table S5 – left and middle panels).

Supplementary figures

Supplementary Figure S1: *Interpretation and visualization of the most important coefficients of logistic mixed regression models predicting accuracy duration as a function of the Visibility rating.*

X axis *represents the Visibility rating centred on the lowest rating. The point on the scale corresponds to the following Visibility ratings: 0 – no experience ('I saw nothing'), 1 – vague experience ('I saw a glimpse'), 2 – almost clear experience ('I saw almost clearly'), 3 – clear experience ('I saw clearly').* ***Y axis*** *represents logit of discrimination accuracy.* ***Logit of 0*** *corresponds to a chance level of performance (in the case of discrimination between two possible options – 50% of discrimination accuracy).* ***Coefficient interpretation.*** *When fitting a logistic mixed regression model predicting accuracy as a function of the Visibility rating, we should interpret output coefficients as follows (red numbers on the plot): 1. Intercept – accuracy at the lowest Visibility rating – subjective visibility criterion. 2. Visibility rating – regression slope – the relation between discrimination accuracy (T1 response) and subjective visibility (T2 response). The slope represents metacognitive accuracy. The steeper the slope, the better the metacognitive accuracy. If other factors are added to the model, in order to compare metacognitive accuracy we should check coefficients representing the Visibility rating's (PAS) interaction with the additional factor we introduced to the model. For example, if we want to check how metacognitive accuracy differs between Conditions, we should check the coefficient representing Visibility rating (PAS) * Condition interaction.*

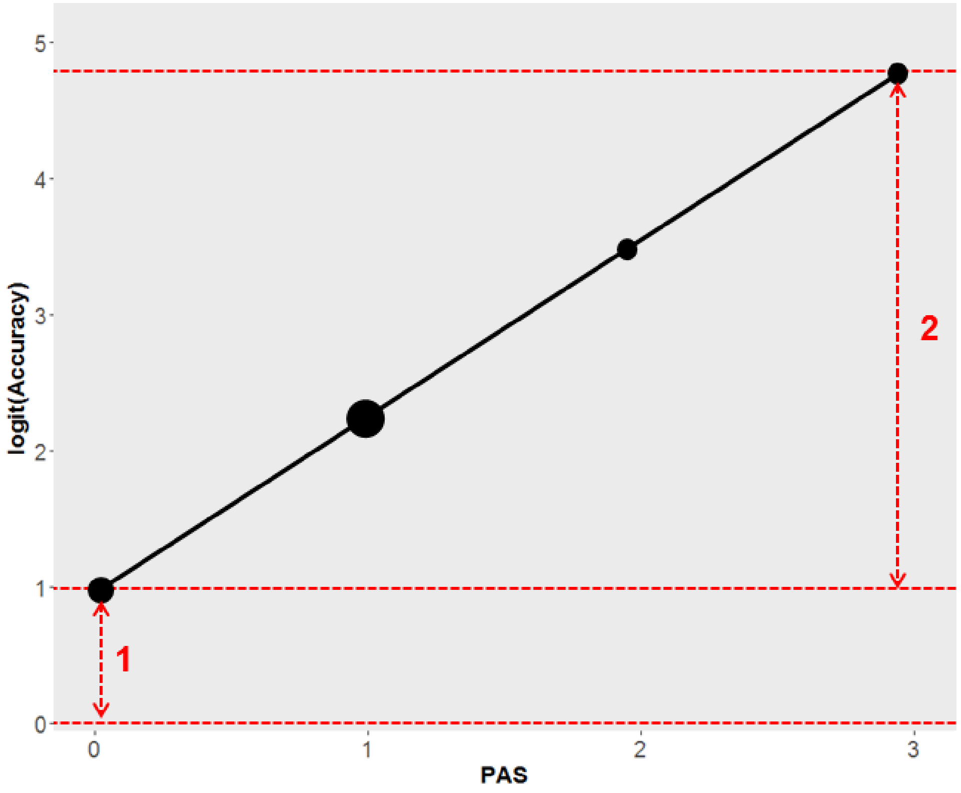

Supplementary Figure S2: *Interpretation and visualization of the most important coefficients of linear mixed regression models predicting changes in IBI duration as a function of Time and Accuracy.*
***X axis** represents the 3000 ms interval between T1 response and T2 scale presentation expressed either in ordinal Epoch values or continuous Time in ms. **Y axis** represents predicted values of baseline-corrected IBI values in ms. Pre-stimulus cardiac activity serves as a baseline (see: ECG pre-processing). The **solid line** represents correct and the **dotted line** – incorrect trials. The horizontal **red line at Y = 0** delineates heartbeat acceleration from deceleration. If IBI change falls below the line, it means the heart decelerated and was beating slower (i.e. IBIs were longer) than the pre-stimulus baseline. If IBI change rises above the line, it means the heart accelerated and was beating faster (i.e. IBIs were shorter) than the pre-stimulus baseline. **Coefficient interpretation.** When fitting a linear mixed regression model predicting IBI change as a function of T1 response Accuracy and Time, we should interpret output coefficients as follows (red numbers on the plot): 1. Intercept = IBI change in 300 ms following T1 response in Error trials. 2. Accuracy = accuracy-dependent difference (i.e. difference between Error and Correct trials) in IBI change in 300 ms (i.e. first Epoch) following T1 response. 3. Epoch/Time = dynamic (slope) of cardiac activity following T1 response in Error trials. 4. Accuracy * Epoch/Time = accuracy-dependent difference in Time/Epoch slopes = accuracy-dependent difference in the dynamic of cardiac activity following T1 response = information about discrimination accuracy coded in cardiac activity. If other factors are added to the model, in order to compare accuracy-dependent difference in the dynamic of cardiac activity following T1 response (i.e. the amount of information about discrimination accuracy coded in cardiac activity), we should check coefficients representing Accuracy * Epoch/Time interaction with the additional factor we introduced to the model. For example, if we want to check how an accuracy-dependent difference in the dynamic of cardiac activity following T1 response changes with increasing visibility (i.e. increasing Visibility – PAS – rating), we should check the coefficient representing Accuracy * Epoch/Time * PAS interaction. Another example, if we want to check how an accuracy-dependent difference in the dynamic of cardiac activity following T1 response differs between Conditions, we should check the coefficient representing Accuracy * Epoch/Time * Condition interaction.*

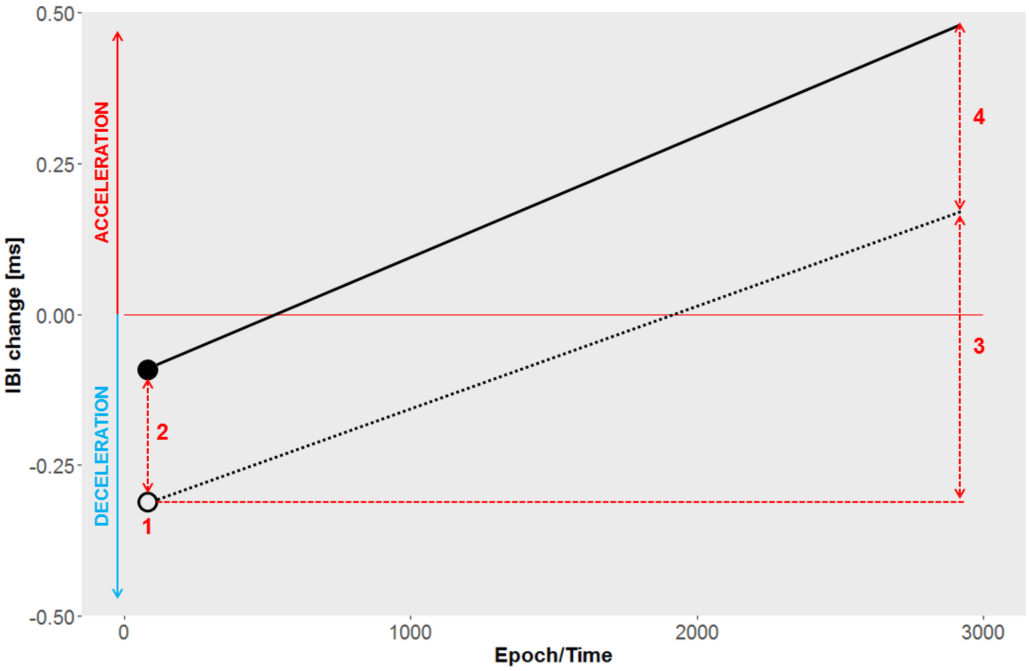

Supplementary Figure S3: *Individual differences in metacognitive accuracy. Results of the logistic regression model fitted for each participant (columns) separately in Experiment 1 (a) and in Experiment 2 (b) with additional comparison between real and fake cardiac feedback.*

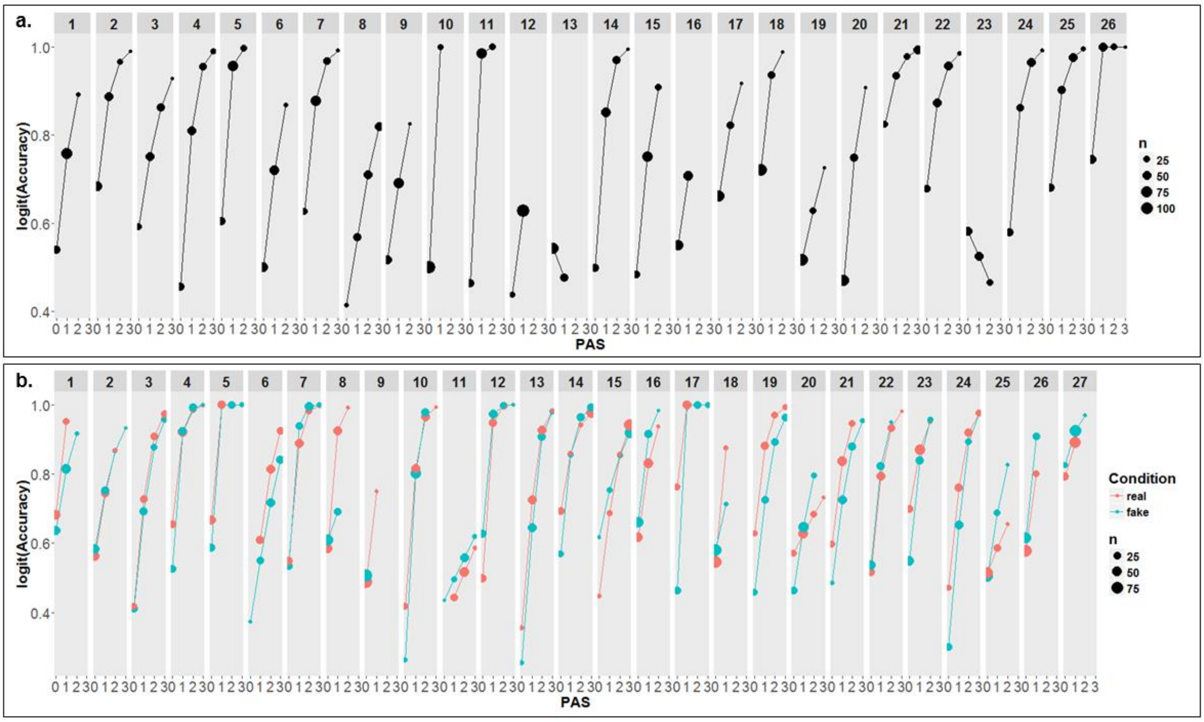

Supplementary Figure S4: *Individual heart rate and its variability. Average inter-beat interval for each subject a. in Experiment 1 and b. in Experiment 2 with between-condition comparison. Error bars represent within-subject standard deviation of IBI.*

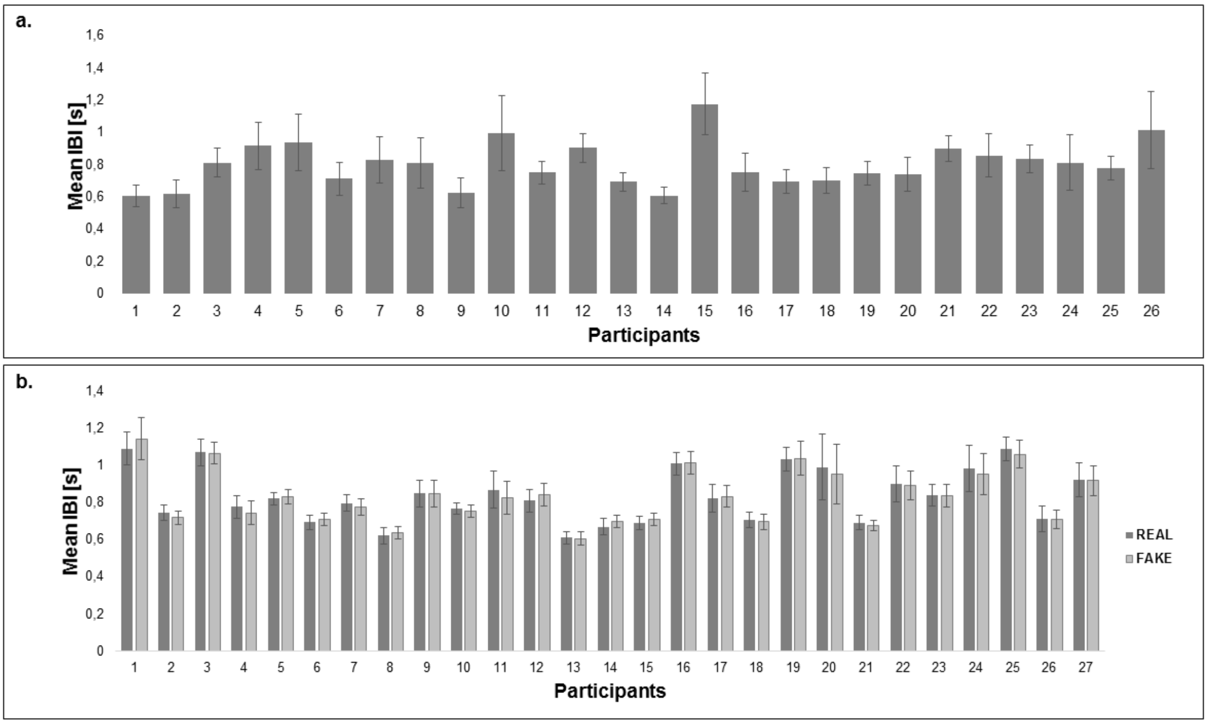

Supplementary Figure S5: *Group means of IBI. Error bars represents standard deviation.*

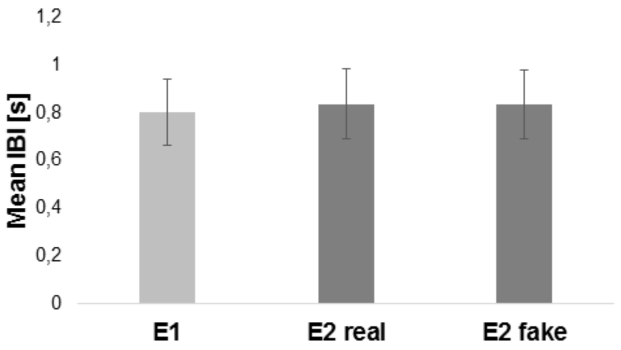

Supplementary Figure S6: *Type-1-response-related changes in IBI duration with respect to Epoch, Accuracy, Visibility rating in Experiment 1 (a) and 2 (b), and depending on cardiac feedback Condition in Experiment 2 (c). Y-axis represents raw IBI changes.*

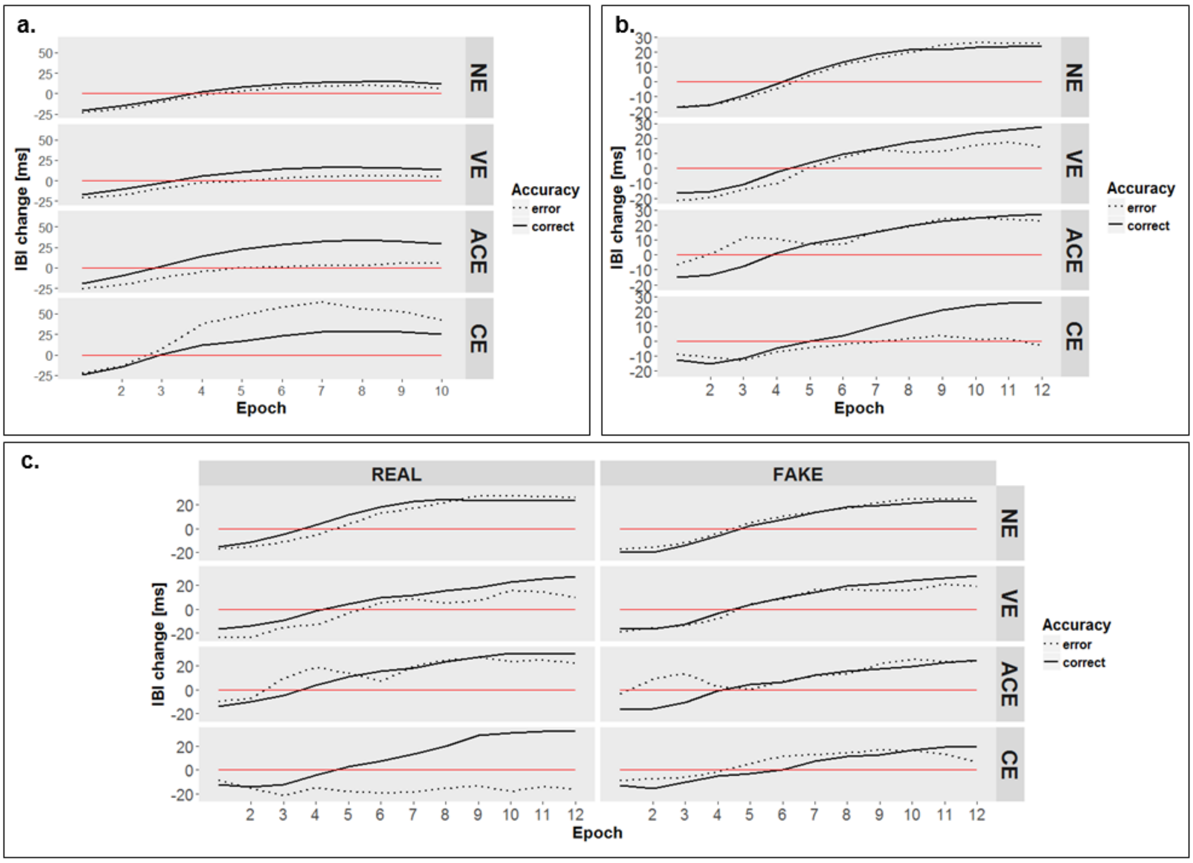

Supplementary tables

Supplementary Table S1: *Regression coefficients for the logistic regression mixed model for accuracy in each Presentation Time in Experiment 1. The model was centred on the lowest Visibility rating. PAS (Perceptual Awareness Scale) slopes represent metacognitive accuracy, i.e. how well PAS ratings predict accuracy.*

	Estimate	SE	z	p	
16 ms	.21	.14	1.45	.146	
32 ms	.57	.16	3.53	.001	***
48 ms	.33	.17	1.91	.056	.
64 ms	.69	.2	3.51	.001	***
16 ms: PAS	.47	.15	3.21	.001	**
32 ms: PAS	.71	.13	5.36	.001	***
48 ms: PAS	1.29	.16	8.25	.001	***
64 ms: PAS	.99	.14	6.99	.001	***

N = 26 # observations = 3181

*. p < .1, * p < .05; ** p < .01; *** p < .001*

Supplementary Table S2: *Regression coefficients for the logistic regression mixed model for accuracy in each Presentation Time in Experiment 2. The model was centred on the lowest Visibility rating in the real Condition. So, the reference Condition is the real cardiac feedback. PAS (Perceptual Awareness Scale) slopes represent metacognitive accuracy, i.e. how well PAS ratings predict accuracy. Condition * PAS slope represents the difference in metacognitive accuracy between conditions.*

	Estimate	SE	z	p	
16 ms	.06	.16	.39	.69	
32 ms	.31	.17	1.82	.067	.
48 ms	.63	.21	3.06	.002	**
64 ms	.79	.24	3.34	.001	***
16 ms: PAS	.48	.13	3.72	.001	***
32 ms: PAS	.89	.13	6.56	.001	***
48 ms: PAS	1.32	.17	7.62	.001	***
64 ms: PAS	1.16	.17	6.77	.001	***
16 ms: Condition	-.09	.13	-.61	.538	
32 ms: Condition	-.19	.17	-1.07	.281	
48 ms: Condition	.2	.22	.88	.375	
64 ms: Condition	-.31	.27	-1.11	.264	
16 ms: Condition: PAS	-.07	.16	-.43	.662	
32 ms: Condition: PAS	.09	.18	.46	.642	
48 ms: Condition: PAS	-.39	.22	-1.76	.078	.
64 ms: Condition: PAS	.23	.23	.98	.324	

N = 27 # observations = 4954

*. p < .1, * p < .05; ** p < .01; *** p < .001*

Supplementary Table S3: *Regression coefficients for the linear regression mixed model for IBI change in both experiments fitted within Visibility ratings. Left panel represents intercept and slopes for Experiment 1, right panel for Experiment 2.*

	Experiment 1					Experiment 2				
	Estimate	SE	*z*	*p*		Estimate	SE	*z*	*p*	
NE	-15.250	4.546	-3.355	.001	***	-18.449	4.543	-4.061	.001	***
VE	-14.603	4.288	-3.406	.001	***	-20.119	4.826	-4.169	.001	***
ACE	-20.508	7.750	-2.646	.008	**	-5.220	6.474	-.806	.420	
CE	6.838	16.237	.421	.674		1.343	14.908	.090	.928	
NE: Epoch	3.613	.331	10.911	.001	***	4.546	.236	19.302	.001	***
VE: Epoch	3.080	.460	6.697	.001	***	3.810	.348	10.949	.001	***
ACE: Epoch	3.391	1.160	2.923	.004	**	2.553	.660	3.871	.001	***
CE: Epoch	8.368	2.908	2.877	.004	**	1.225	1.287	.952	.341	
NE: Accuracy	3.207	2.345	1.368	.171		4.355	2.030	2.145	.032	*
VE: Accuracy	3.141	2.813	1.116	.264		4.680	2.566	1.824	.068	
ACE: Accuracy	5.280	6.791	.777	.437		-7.634	4.693	-1.627	.104	
CE: Accuracy	-22.656	16.150	-1.403	.161		-14.073	8.706	-1.616	.106	
NE: Epoch: Accuracy	.290	.437	.663	.507		-.390	.312	-1.251	.211	
VE: Epoch: Accuracy	.427	.520	.822	.411		.566	.391	1.449	.147	
ACE: Epoch: Accuracy	2.352	1.216	1.933	.053	.	1.617	.697	2.319	.020	*
CE: Epoch: Accuracy	-2.654	2.985	-.889	.374		3.053	1.323	2.307	.021	*

N = 26 # observations = 31829 *N = 27 # observations = 59271*

*. p < .1; * p < .05; ** p < .01; *** p < .001*

Supplementary Table S4: *Regression coefficients for the regression mixed model for IBI change in both experiments with reaction time as the additional fixed factor. Left panel represents intercept and slopes for Experiment 1, right panel for Experiment 2.*

	Experiment 1					Experiment 2				
	Estimate	SE	*z*	*p*		Estimate	SE	*z*	*p*	
Intercept	-13.98	4,2	-3.33	.001	***	-27.4	4.71	-5.81	.001	***
Epoch	3.48	.32	10.85	.001	***	4.59	.23	20.40	.001	***
PAS	-.16	2.27	-.07	.943		3.98	2.09	1.9	.057	.
Accuracy	4.08	2.17	1.88	.06	.	5.55	1.85	3	.003	**
RT	-.01	0	-.19	.848		.01	0	7.9	.001	***
Epoch: PAS	-.04	.42	-.09	.929		-.96	.25	-3.77	.001	***
Epoch: Accuracy	-.06	.4	-.14	.885		-.36	.28	-1.26	.208	
PAS: Accuracy	-2.08	2.46	-.85	.398		-3.51	1.83	-1.91	.056	.
Epoch: PAS: Accuracy	.8	.46	1.76	.079	.	.97	.28	3.51	.001	***

N = 26 # observations = 31829 *N = 27 # observations = 59271*

Supplementary Table S5: *Between conditions comparison of regression coefficients for the regression mixed model for IBI change in Experiment 2, with reaction time as the additional fixed factor. Left panel represents intercept and slopes for the real cardiac feedback condition, middle panel for the fake cardiac feedback, and right for comparison of between condition coefficients.*

	Real cardiac feedback					Fake cardiac feedback					Condition comparison				
	Estimate	SE	*z*	*p*		Estimate	SE	*z*	*p*		Estimate	SE	*z*	*p*	
Intercept	-24.74	5	-4.95	.001	***	-29.68	4.96	-5.99	.001	***	.32	2.93	.11	.914	
Epoch	4.76	.33	14.59	.001	***	4.45	.31	14.28	.001	***	-.31	.45	-.69	.489	
PAS	2.34	2.73	.86	.391		5.48	2.62	2.09	.036	*	3.24	3.31	.98	.328	
Accuracy	9.53	2.64	3.61	.001	***	1.58	2.58	.62	.538		-8.33	3.68	-2.27	.023	*
RT	.01	.01	4.06	.001	***	.01	.01	8.07	.001	***	.01	.01	7.64	.001	***
Epoch: PAS	-1.13	.37	-3.05	.002	**	-.81	.35	-2.30	.021	*	.32	.51	.63	.531	
Epoch: Accuracy	-.96	.41	-2.36	.018	*	.24	.40	.60	.547		1.19	.57	2.11	.035	*
PAS: Accuracy	-4.37	2.63	-1.66	.097	.	-2.53	2.52	-1.01	.315		1.07	3.61	.3	.768	
Epoch: PAS: Accuracy	1.49	.40	3.72	.001	***	.47	.38	1.22	.221		-1.02	.56	-1.84	.066	.

N = 27 # observations = 59271

*. p < .1, * p < .05; ** p < .01; *** p < .001*

Supplementary Table S6: *Manipulation check – cardiac feedback. Only 9 out of 27 participants noticed any difference between real and fake cardiac feedback. In both real and fake conditions, the majority claimed that the auditory feedback reflected their own cardiac activity.*

	Difference	Real	Fake
Yes	9	22	19
No	18	5	7

References

1. Schandry, R. Heart beat perception and emotional experience. *Psychophysiology* **18,** 483–488 (1981).

2. Garfinkel, S. N., Seth, A. K., Barrett, A. B., Suzuki, K. & Critchley, H. D. Knowing your own heart: Distinguishing interoceptive accuracy from interoceptive awareness. *Biol. Psychol.* **104,** 65–74 (2015).

3. Porges, S. *Body perception questionnaire: Laboratory of development assessment*. (University of Maryland, 1993).

4. Łukowska, M., Sznajder, M. & Wierzchoń, M. The relationship between access to interoceptive information and performance in the backward masking task.

5. Sznajder, M. & Łukowska, M. Python Online and Offline ECG QRS Detector based on the Pan-Tomkins algorithm [Data set]. *Zenodo* (2017). doi:10.5281/zenodo.826614

6. Pan, J. & Tompkins, W. J. A Real-Time QRS Detection Algorithm. *IEEE Trans. Biomed. Eng.* **BME-32,** 230–236 (1985).

7. Butterworth, S. On the theory of filter amplifiers. *Wirel. Eng.* **7,** 536–541 (1930).

8. Berntson, G. G. *et al.* Heart rate variability: Origins, methods, and interpretive ceveats. *Psychopsysiology* 623–648 (1997). doi:10.1111/j.1469-8986.1997.tb02140.x

9. Hajcak, G., McDonald, N. & Simons, R. F. To err is autonomic: Error-related brain potentials, ANS activity, and post-error compensatory behavior. *Psychophysiology* **40,** 895–903 (2003).

10. Binder, M., Barry, R. J. & Kaiser, J. Sensitivity of primary phasic heart rate deceleration to stimulus repetition in an habituation procedure: influence of a subjective measure of activation/arousal on the evoked cardiac response. *Int. J. Psychophysiol.* **55,** 61–72 (2005).
